# Cement Composites with Graphene Nanoplatelets and Recycled Milled Carbon Fibers Dispersed in Air Nanobubble Water

**DOI:** 10.3390/nano12162786

**Published:** 2022-08-14

**Authors:** Anastasia I. Patrinou, Eirini Tziviloglou, Athanasios Varoutoglou, Evangelos P. Favvas, Athanasios C. Mitropoulos, George Z. Kyzas, Zoi S. Metaxa

**Affiliations:** 1 Department of Chemistry, International Hellenic University, 65404 Kavala, Greece; 2 Research Unit of Advanced Materials, Department of Financial Engineering, School of Engineering, University of the Aegean, 41 Kountouriotou Str., 82132 Chios, Greece; 3 Institute of Nanoscience and Nanotechnology, NCSR “Demokritos”, 15341 Agia Paraskevi, Greece

**Keywords:** graphene nanoplatelets, recycle carbon fibers, air nanobubbles, cement-based composites and nanocomposites, mechanical properties, electrical properties

## Abstract

The individual effect of nano- and micro-carbon-based fillers on the mechanical and the electrical properties of cement paste were experimentally examined in this study. The objective of the study was to separately examine the effects of size and morphology (platelets and fibers) of nano- and micro-reinforcement. Three different sizes of Graphene Nanoplatelets (GNPs), at contents of 0.05% and 0.20% and recycled milled carbon fibers (rCFs), at various dosages from 0.1–2.5% by weight of cement, were incorporated into the cementitious matrix. GNPs and rCFs were dispersed in water with air nanobubbles (NBs), an innovative method that, compared to common practice, does not require the use of chemicals or high ultrasonic energy. Compressive and bending tests were performed on GNPs- and rCFs-composites. The four-wire-method was used to evaluate the effect of the conductive fillers on the electrical resistivity of cement paste. The compressive and flexural strength of all the cementitious composites demonstrated a considerable increase compared to the reference specimens. Improvement of 269.5% and of 169% was observed at the compressive and flexural strength, respectively, at the GNPs–cement composites incorporating the largest lateral size GNPs at a concentration of 0.2% by weight of cement. Moreover, the rCFs–cement composites increased their compressive and flexural strength by 186% and 210%, respectively, compared to the reference specimens. The electrical resistivity of GNPs- and rCFs-composite specimens reduced up to 59% and 48%, respectively, compared to the reference specimens, which proves that the incorporation of GNPs and rCFs can create a conductive network within the cementitious matrix.

## 1. Introduction

Recognizing cement as one of the dominant materials of the construction industry, researchers from around the world focus on optimizing its structural health. Cement-based materials have been extensively used due to their high availability and low construction and maintenance costs. However, their brittleness and low tensile strength may significantly decrease their functionality [1]. Cement-based materials are prone to cracking, which might affect the integrity of structures over time [2]. The presence of cracks can cause cement degradation by increasing its permeability, and might make cement vulnerable to corrosion, chloride penetration, etc., leading to accelerated deterioration [3]. Therefore, it is important to enhance the cracking resistance of cement-based materials. Reinforcement of cementitious composites in micro- and macro- scale using fibers and other fillers is a common method for the improvement of the intrinsic brittle behavior of cement-based materials [4]. Nano- and micro-reinforcement can increase the tensile strength of the cement matrix by enhancing the load-transfer capacity and the crack bridging mechanism. 

Recent advances in materials science and nanotechnology enable the use of various types of carbon-based nanomaterials, such as nanofibers, nanotubes, and nanoparticles, as reinforcing agents in the cement to control the creation of cracks at the nanoscale. Carbon nanomaterials demonstrate outstanding mechanical chemical and physical properties, such as low density, high specific surface area, hardness, and excellent chemical and thermal stability. Carbon nanomaterials incorporated in cement-based materials at relatively low proportions provide important improvement in the compressive, tensile, and flexural strength [5,6,7], as well as new functions such as stress- and strain-sensing, temperature monitoring, and energy harvesting [8,9,10,11].

Lately, graphene-based materials have captured the attention of scientists. Graphene has extraordinary thermal, electrical, and mechanical properties, and it has been described as the most powerful, tough, thin, and dense material in the world [12]. Both its electrical and thermal conductivity are considered superior to the materials used to date. Recently, a number of graphene composites have been developed utilizing the excellent properties of graphene [13]. Graphene Nanoplatelets (GNPs) are of particular interest for combining superior properties, such as two-dimensional planar structure, great aspect ratio, remarkable strength and durability, thermal, electrical, and chemical stability, with low cost and weight, and large-scale production potential. They are also electrical and thermal conductors [14]. Compared to pristine graphene, GNPs are a promising alternative as nanofillers in material science, due to their availability in the market and their affordable prices [15]. 

By incorporating GNPs in cement to develop innovative and advanced composites, improvements in the stability and longevity of structures are possible [16]. Research has shown that the use of GNPs accelerates the hydration reactions of cement, improves the porosity, and creates a denser microstructure [17,18]. It has also been reported that GNPs are able to increase the compressive and flexural strength of the matrix. Silva et al. [19] found that the incorporation of GNPs in mortar, in a small dosage 0.021% by weight, enhanced the compressive strength by 95.7% after 28 days. Tong et al. [20] used 0.1% by weight GNPs and recorded 19.9% increase in the compressive strength. Other studies refer that cement composites modified with GNPs 0.05% and 0.06% by weight, show flexural strength increase of 15–24% and 27.8%, respectively [21,22]. GNPs have also been used to create conductive cement-based composites, which can monitor their own strain by detecting alterations in the electrical resistivity values [23,24]. 

Natural and synthetic fibers are widely used in fiber-reinforced cement-based materials [25]. The material, the type, and the amount of the fibers used determine the properties of the composite materials [26]. The implementation of microfibers can greatly improve the mechanical strength of cement and restrain the crack growth into the matrix, however, they cannot prevent crack formation [27]. Due to their unique properties, carbon fibers stand out compared to other types of fibers. Carbon fibers (CFs) have many advantages, including excellent strength-to-weight ratio, high tensile strength and stiffness, low weight, chemical and thermal stability, and low thermal expansion [28,29]. They have high electrical conductivity and can be used to reduce the electrical resistance and provide self-detecting capabilities, as well as to create a shield of electromagnetic protection in building materials [30]. An increase of 85% in flexural strength and 22% in compressive strength has been reported, along with the introduction of a small amount of CFs (0.189% by volume) into concrete [31]. Cholker et al. [32] studied the behavior of “smart” carbon reinforced concrete with microfibers. The specimens were assessed under loading-unloading cycles to examine the stress and damage sensing ability. It was derived that the presence of CFs in 1.5% and 2% by weight of cement provides a sufficient link between stress and strain of concrete and electrical resistivity. In other research, it was found that cement-based composites reinforced with short CFs are capable of sensing their own damage, stress, and temperature [33].

CFs, despite their numerous advantages in modifying the performance of cement composites, have excessive cost, which is a deterrent to their use. Nowadays, the use of recycled CFs (rCFs) as reinforcing material has gained increasing attention owing to their durability and outstanding properties with decreased cost [34]. rCFs reveal superior mechanical performance, attributed to their rugged surface, which creates a stronger bond with the surrounding cement matrix [35]. Faneca et al. [36] used rCFs to fabricate low cost electrically conductive cement composites that can be employed not only in laboratory scale, but also in the civil engineering industry. Akbar and Liew [37] investigated the effect of elevated temperatures on the reinforcement mechanism of cement composites containing rCFs. The amount of fibers and elevated temperatures remarkably affect the mechanical properties and the mass loss of the rCFs–cement composites. Mastali and Dalvand incorporated rCFs 1.25% by volume in plain concrete, which resulted in an increase of 65.10% and of 66.93% on the compressive and the flexural strength of specimens, respectively. Moreover, the impacted resistance increased 6.48 times with rCFs concentration of 1.25% [38]. rCFs represent an alternative to obtain superior performance and low-cost cementitious composites, and they are commercially available by several companies. However, the scientific research on rCFs–cement composites has been limited. The effect of rCFs on the mechanical properties, as well as the interaction between the rCFs and the surrounding cement matrix, has not been investigated yet.

The homogeneous dispersion of carbon materials in cementitious composites is another critical issue that strongly influences the reinforcing efficiency and the ultimate performance of the cement composites. GNPs and rCFs, due to strong Van der Waals forces that are developed among them, have the tendency to agglomerate. Their hydrophobic nature makes their homogeneous dispersion in aqueous suspensions challenging [39]. However, homogeneous dispersion is usually succeeded either mechanically, by using high ultrasonic energy, or chemically, by using various water-reducing agents, such as superplasticizers as surfactants [40,41,42]. A new promising technology called ‘nanobubble technology’ is utilized by many important fields for applications, including flotation, nanomaterials dispersion, and crystal growth [43,44,45]. Nanobubble nucleation is favored at hydrophobic particle surfaces, enhancing the stability of micro-bubble suspensions, and thus, facilitating particle flotation [46]. The use of water enriched with air nanobubbles improves the morphological characteristics of the dispersed nanoparticle clusters without the presence of aggregates [45]. To our knowledge, the application of this technology on dispersing nano- and micro-carbon materials for use in cement pastes has not been previously studied. 

Recently, extensive research has been conducted on the improvement of the mechanical and electrical properties of cementitious composites using carbon-based materials. Their size and morphology strongly affect their enhancing action. However, the study on the reinforcing mechanism of different fillers based on their dimensions or size scales is narrow. The influence of the different type and scale of the reinforcing materials on the mechanical and the electrical properties of cement-based composites has not been explicitly clarified yet. 

In this research, two commercially available carbon-based materials, GNPs and milled rCFs, were incorporated in the cementitious matrix to serve as reinforcement in nano- and micro-scale. Composite mixtures with three different sized GNPs, at contents of 0.05% and 0.20% and rCFs at proportions from 0.1–2.5% by weight of cement, were fabricated. The prepared specimens were assessed for their compressive strength, flexural strength and electrical resistivity. Overall, recycled milled carbon fibers with improved carbon footprint and lower cost were investigated in the present study, as they differ significantly in size from short carbon fibers reported in the literature (rCFs length is at the μm scale while typical CFs have length at the mm scale) and the research of their effect on the mechanical properties of cement-based materials is limited. Another innovative aspect of this work is the usage of water with air nanobubbles (NBs) for the proper dispersion of GNPs and rCFs. It is noteworthy that compared to frequently used practices, this method does not require the use of chemicals or high ultrasonic energy, and it can be easily applied on the construction site. 

## 2. Materials and Methods

### 2.1. Materials and NBs Method

A commercial Portland cement CEM II 32,5 N (TITAN Cement Company S.A Athens, Greece) was used for this study. Three types of GNPs were used, namely N008-100-N, N008-100-P10, N008-100-P40 (Angstron Materials Inc. Dayton, OH, USA), exhibiting the same thickness but different lateral size (diameter). The physical properties of the GNPs are shown in Table 1. Moreover, milled rCFs, produced by Haufler Composites GmbH & Co. KG, Blaubeuren, Germany, were utilized in this study. According to the manufacturer CF−milled100 is a mixture of all origins carbon and graphite ex-PAN fibers, obtained from virgin carbon fibers, chosen and milled for compounding. Their mechanical and physical properties are shown in Table 2.

**Figure 1:**
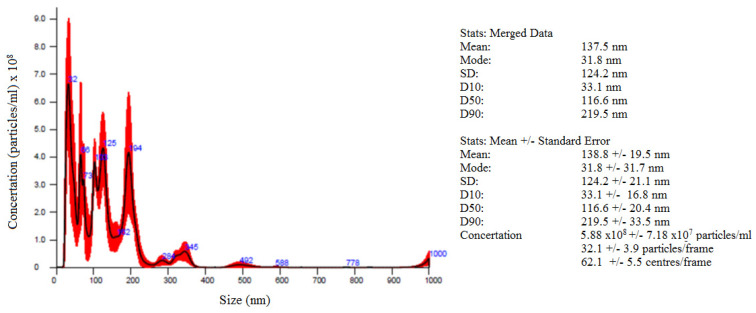
Nanobubbles concentration versus size.

**Figure 2:**
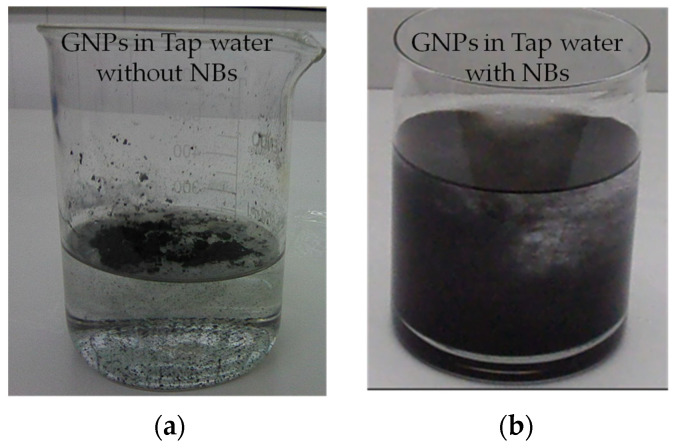
GNP suspensions in (**a**) plain tap water and (**b**) tap water with nanobubbles.

All specimens were prepared with tap water (low salinity, S < 350 mg/L) enhanced with air nanobubbles (NBs). Air nanobubbles were created using a generator which utilized the counterflow hydrodynamic cavitation. Details about the used NBs generator can be found in our previous work [47]. The mean size and the concentration of the produced NBs are shown in Figure 1. The optimal processing time, regarding the size of the produced NBs, was 30 min [47]. The NBs methodology was chosen for the adequate dispersion of the GNPs and rCFs. Due to the efficacy of the method, no additives, such as superplasticizers, water reducing agents, or surfactants, were employed in this study. Another advantage of this method is that zero energy consumption is required, as the water feed pressure must be at 2.5 bar, equal to the water supply network pressure, and of course it can be easily applied in the cement industry. The difference in the dispersion quality between aqueous solutions with and without NBs is easily noticeable (Figure 2). 

### 2.2. Preparation of Composites Specimens

**Figure 3:**
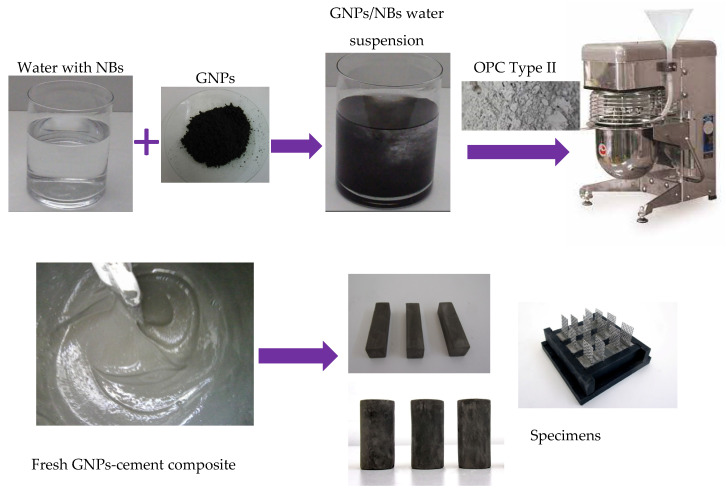
GNP–cement composite preparation.

Two concentrations of each GNPs-type were used, namely 0.05% and 0.2% by weight (wt.) of cement. Furthermore, six mixtures of milled rCFs were prepared at different concentrations, namely 0.0%, 0.1%, 0.5%, 1.0%, 1.5%, 2.0%, 2.5% by weight of cement. The reinforcing materials were added into water with NBs, and the aqueous suspensions were then poured in the mixer (Figure 3). The pastes were prepared in a stainless-steel standard mixer, according to ASTM C305, at low speed (140 rpm) for 30 s and then at high speed (285 rpm) for 60 s. The water-to-cement ratio (w/c) was constant at 0.50 for all the mixtures. The proportions of the materials used for reference, GNPs- and rCFs-composites are presented in Table 3 and Table 4, respectively.

**Figure 4:**
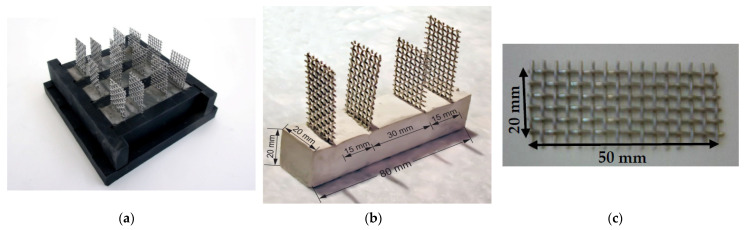
Specimens for the electrical resistance tests (**a**) inside the mold write after casting, (**b**) after demolding, and (**c**) stainless steel mesh embedded inside the samples.

The prepared GNPs- and rCFs–cement composites were cast in 30 mm × 60 mm cylindrical steel molds and 20 mm × 20 mm × 80 mm prismatic plastic molds, to investigate the compressive and flexural strength, respectively. To measure the electrical resistance of the composite specimens, right after molding, four stainless steel electrodes were embedded into the specimens covering their entire cross-section (Figure 4a,b). The stainless steel mesh used had openings of 1.74 mm × 1.74 mm, wire thickness of 0.8 mm, and dimensions of 20 mm × 50 mm (Figure 4c). After 48 h, the specimens were de-molded and placed in tap water tanks with calcium hydroxide, where they were kept for 28 days.

### 2.3. Experimental Measurements

**Figure 5:**
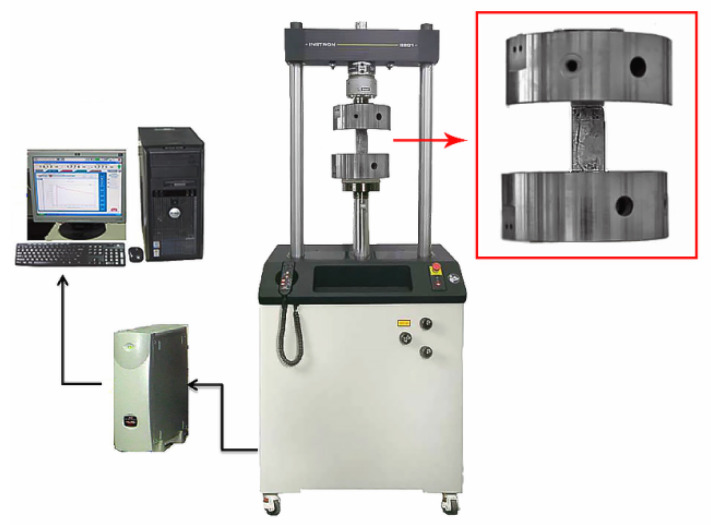
Experimental apparatus for compressive stress test (close up shows a fractured sample).

The effect of GNPs and milled rCFs on the compressive strength pastes was evaluated after 28 days of curing. Three specimens of each testing series were assessed. The compressive strength was measured using the Instron 8801 testing machine (Instron, Norwood, MA, USA) with maximum load capacity of 100 kN at a constant displacement rate of 0.2 mm/min. Loading was applied monotonically until the failure of the specimen (Figure 5).

**Figure 6:**
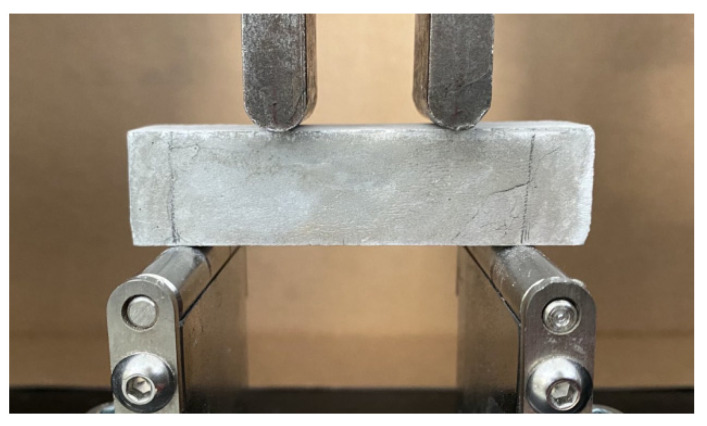
4-point bending test experimental set up.

The effect of GNPs and milled rCFs on the flexural strength of the cement paste was evaluated after 28 days of curing. Flexural strength measurements were carried in a 4-point-bending configuration (Figure 6) using an MTS Insight testing machine (MTS Systems Corporation, Eden Prairie, MN, USA) with maximum load capacity of 10 kN applying a constant displacement rate of 0.002 mm/min (Figure 4b). The average flexural strength of three specimens was recorded and presented as representative flexural strength of the cement composites.

**Figure 7:**
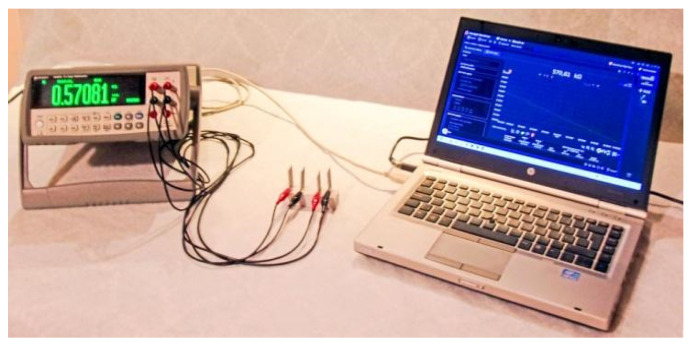
Four-wire method set-up.

The electrical resistance was measured using the four-wire-method (Figure 7). Three specimens of each testing series were tested. After 28 days of curing, the specimens were placed in a laboratory drying oven where they remained for 72 h, at a temperature of 80 °C, to evaporate the water trapped in the pores. A 34450A Keysight Laboratory Digital Bench Multimeter, Keysight Technologies, Santa Rosa, CA, USA )was used for the electrical measurements. The internal electrodes were used to measure the voltage, while the external electrodes were used to supply the current as shown in Figure 7.

During the measurements, a rubber material was placed under the specimens to insulate the specimen. The electrical resistance was recorded every 2 s over a period of 30 min to avoid potential deviations caused by the effects of electric polarization. Data from the last five minutes of the measurements were used to calculate the average resistance values. The resistivity, *ρ*, of the nanocomposites was calculated using Ohm’s law as

$$\rho =R\frac{S}{L}$$

where *R* is the electrical resistance, *S* is the cross-section of the sample, and *L* is the distance between internal electrodes.

## 3. Results and Discussion

### 3.1. GNPs Strenthening Mechanism

**Figure 8:**
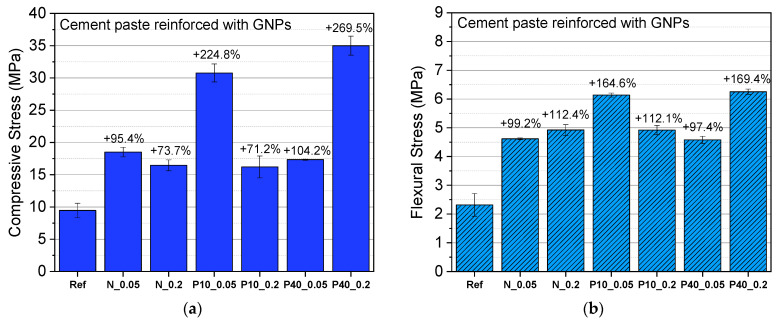
Average (**a**) compressive strength and (**b**) flexural strength of cement composites with GNPs at 28 d.

The average measured values of compressive and flexural strength of cement composites with different sizes and contents of GNPs are illustrated in Figure 8. The abbreviations of N, P-10, and P-40 shown in the graph refer to the N008-100-N, N008-100-P10, and N008-100-P40 GNPs types followed by their concentration 0.05 and 0.2% by weight of cement, i.e., P10_0.05 is the N008-100-P10 GNPs-type at an amount of 0.05%.

The incorporation of GNPs into the cement paste is beneficial for its compressive and flexural strength. The compressive strength of the plain cement paste was found to be 9.5 MPa and raised to 30.8 MPa and 35 MPa for the P10_005 and P40_02 specimens, corresponding to an increase of 224.8% and of 269.5%, respectively, which is more than reported in the literature [19,21,48,49,50]. The GNPs-composites also demonstrated an important increase in the flexural strength compared to the plain cement specimens. The highest flexural strength values varied between 6.14 MPa and 6.25 MPa, corresponding to an increase of 164.6% and 169.4%, for the P10_005 and P40_02 specimens, respectively, compared to the reference specimens (2.32 MPa).

**Figure 9:**
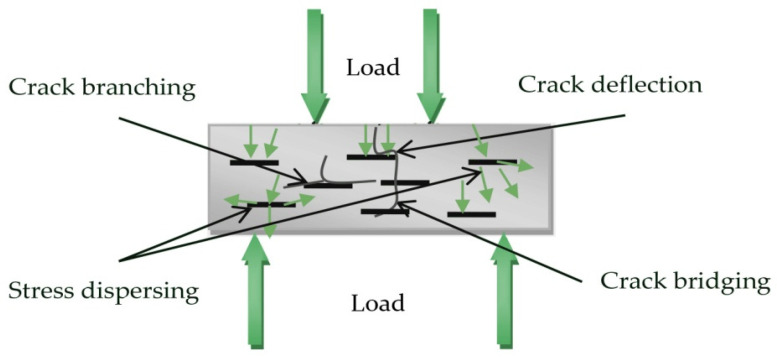
GNPs action under 4-point bending load.

This high improvement in the mechanical properties of GNPs–cement composites can be attributed to the interaction between the GNPs and the paste. GNPs possibly have developed good affinity/bond with the cement matrix, which can enhance the load-transfer capacity by absorbing energy more efficiently and offer a better resistance to crack spreading [17,22,51,52]. Upon load application, GNPs effectively transfer the stress by enhancing the crack bridging mechanism and restraining the crack formation [50,53]. Cracks are blocked and diverted or branched (Figure 9) [19].

According to the results, there were two reinforcing mechanisms that were developed and associated with the GNPs size. When GNPs with a low average diameter of 5 and 10 μm are incorporated, a greater increase in the compressive strength of cement composites is observed at low concentration (0.05 wt.%). This agrees with other studies claiming that GNPs demonstrate better performance at lower concentrations, since a more homogeneous GNPs network can be developed at lower concentrations [49,53]. On the contrary, GNPs type N008-100-P40 demonstrated the maximum compressive stress improvement at the higher concentration (0.2 wt.%). The P-40 type demonstrates up to 10 times larger diameter compared to the N and P-10 GNPs-types. This finding is supported by the fact that larger sized nanomaterials are more easy to disperse [54,55]. Therefore, for the P-40 GNPs, the development of a homogeneous network at a larger concentration is feasible.

### 3.2. rCFs Strenthening Mechanism

**Figure 10:**
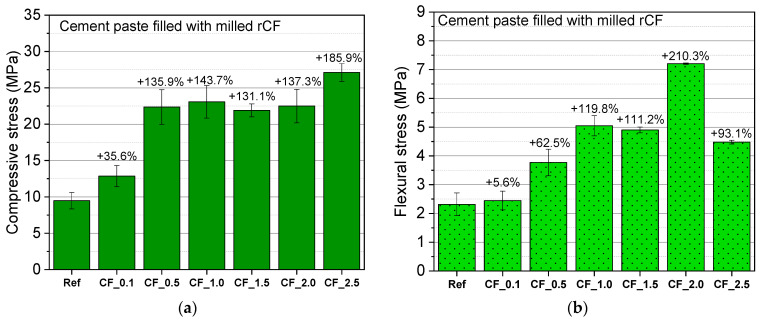
Average (**a**) compressive strength and (**b**) flexural strength of cement composites with milled rCFs at 28 d.

The average compressive and flexural strength values of cement composites reinforced with milled rCFs and their deviation from the reference specimens are shown in Figure 10.

The mechanical strength of cement paste filled with different dosages of rCFs was significantly enhanced. This improvement seems to depend on the rCFs content. The compressive strength of all the specimens was enhanced gradually, with the increase in fiber content ranging from 0.1% to 2.5 wt.%. When the fiber content was 1% and 2.5% by weight of cement, an increase of 143.7% and of 185.9% was recorded, respectively. Similarly, Akbar et al. observed a 47% increase in compressive strength of cement composites by the addition of 1% by volume of milled rCFs [35]. Moreover, Mastali and Dalvand reported 65.10% improvement on the compressive strength of concrete specimens filled with 1.25% by volume rCFs [38].

The rCFs-composites showed considerable improvement in flexural strength compared to the plain cement specimens. A maximum flexural stress of 7.2 MPa was recorded for CF_2.0 mixture (210.3% higher than the reference mixture). This improvement is related to the crack bridging action of CFs. According to the results, the flexural performance of the specimens improves as the fiber content increases. This trend is in line with the findings of Yoo et al. [56], Donnini [30], and Han et al. [57]. As the fiber concentration in the matrix increases, the crack resistance mechanism and the fracture energy absorption improve. However, after a certain concentration, the CFs agglomerate and the mechanical strength of the composite reduces [57].

### 3.3. Electrical Resistivity of GNPs–Cement Composites

**Figure 11:**
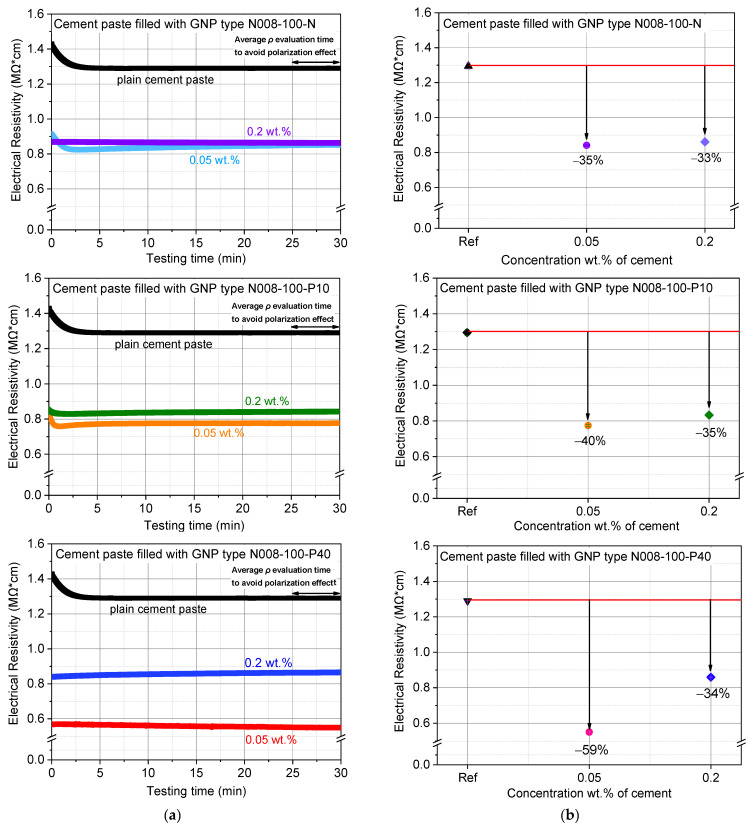
(**a**) Typical electrical resistivity over testing time curves of the GNPs–cement composites; (**b**) Average electrical resistivity results of the GNPs–cement composites.

Typical curves of the electrical resistivity over testing time for the plain cement paste for the three different GNPs-types in both concentrations are shown in Figure 11. High electrical resistivity values (1.29 MOhm·cm) were recorded for the reference mixture. At the beginning of the test, increased values of approximately 1,42 MOhm·cm were observed. This can be explained by the electrical polarization phenomenon. Electric polarization occurs when a dielectric material is exposed to a DC electric field. Next, positive and negative charges are pushed in opposite directions, resulting in separation of positive and negative charges throughout the mass of the material. Consequently, the resistance of the material, especially at the beginning of measurement, is constantly changing. The resistance stabilizes after approximately 3 min; however, marginal scatter of about 5% may be observed in the measurements.

It is evident that GNPs-composites, regardless of the GNPs’ type and amount, exhibit lower resistivity values compared to the plain cement paste [24,49]. The values of the resistivity were lower when the concentration of GNPs was 0.05 wt.%, which may be related to the GNPs dispersion state, i.e., the more uniform the dispersion of GNPs, the lower the electrical resistance in the cement matrix. The lowest *ρ*, 59% reduction compared to the plain cement paste, was measured for the specimens with GNPs-type N008-100-P40. Although the concentration of GNPs remained the same in all specimens, the results indicate that N008-100-P40 created the most efficient conductive network within the cementitious matrix. This is probably associated with its diameter, which was larger (44μm) than the other two types of GNPs. Therefore, the distance between the GNPs is small enough to form conductive paths in the matrix, favoring the passage of the current [17]. A significant decrease in the resistivity of nanocomposites modified with low contents of GNPs has been also reported [51,53].

**Figure 12:**
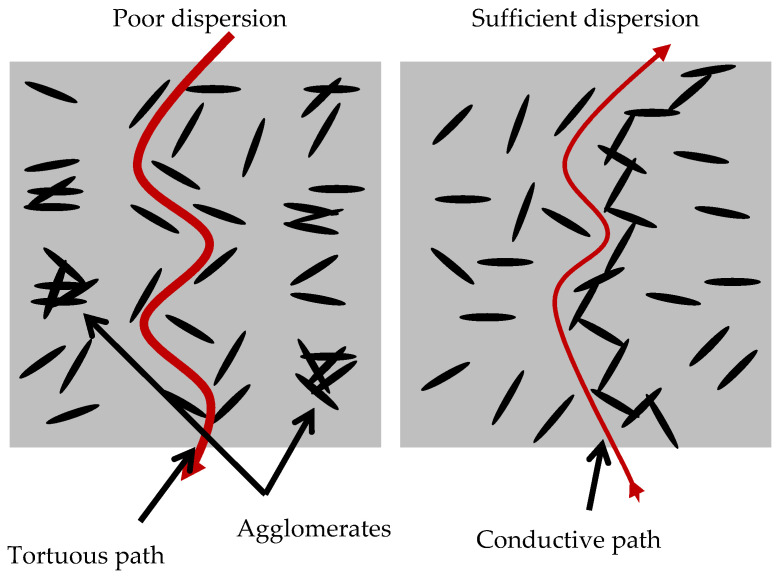
Electrical conductivity of GNPs–cement composites.

When the concentration of GNPs increased to 0.2 wt.%, all the composite specimens demonstrated similar reduction in the resistivity close to 35%. This finding can be attributed to the inadequate dispersion of the nanomaterials. According to the literature [9,58,59], the resistivity of a material depends on the quantity and sufficient dispersion of conductive additives (Figure 12). Inadequate dispersion leads to GNPs-agglomeration, which increases the electrical resistance of the composite.

### 3.4. Electrical Resistivity of rCFs–Cement Composites

**Figure 13:**
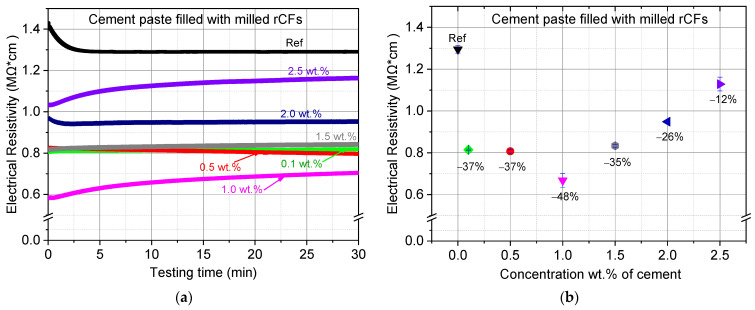
(**a**) Typical electrical resistivity over testing time curves of the investigated rCFs–cement composites; (**b**) Average electrical resistivity results of the GNPs–cement composites.

The typical electrical resistivity curves over testing time for the reference sample, and for rCFs–cement composites, are shown in Figure 13. The rCFs-composites demonstrated reduced resistivity compared to the reference specimens. The rate of resistance reduction is affected by the concentration of rCFs within the matrix. The highest reduction rate of 48% was measured for the specimens with fiber concentration of 1 wt.%. When the fiber content is low, the current flow is difficult, since there might be a large space among the fibers. However, by increasing the concentration of the rCFs, the fibers approach each other, and a stable conductive network is established in the matrix [57]. The value of the resistivity decreases until the percentage of rCFs reaches 1 wt.%, indicating that the percolation threshold, i.e., when the fibers form a continuous electrical network, has been reached. More rCFs cause an increase in the value of the electrical resistance.

## 4. Conclusions

The mechanical and electrical behavior of cement paste reinforced with GNPs and milled rCFs were experimentally examined in this research. The addition of GNPs and rCFs into cement paste had a positive effect on the mechanical and electrical properties.

Among the three types of GNPs used in this study, the most effective proved to be the N008-100-P40 with the larger lateral size. Specimens with 0.2 wt.%. GNPs concentration increased both the compressive and the flexural strength of the cement composites by 269% and 169%, respectively. Furthermore, N008-100-P40 at a content of 0.05 wt.%. created the most effective conductive network within the cementitious matrix, attributed to their larger diameter.

Increasing the content of milled rCFs enhanced the mechanical properties of cement paste. The compressive and flexural strength of the rCFs-composites increased by 186% and 210%, with the incorporation of fibers at contents of 2.5 wt.% and 2 wt.%, respectively. The rate of reduction in electrical resistivity was affected by the concentration of rCFs in the composite. Percolation threshold was reached at a fiber content close to 1 wt.% of cement, as an increase of the fiber dosage was not able to further reduce the electrical resistivity of the cement paste.

## Figures and Tables

**Table 1:** Physical properties of GNPs.

GNP Type	Thickness Z (nm)	Diameter X-Y (μm)	Purity (%)	Density (g/cm^3^)	Specific Surface Area (m^2^/g)
N008-100-N	50–100	<5	98	2.20	13
N008-100-P10	50–100	<10	99	2.20	15
N008-100-P40	50–100	<44	99	2.20	15

**Table 2:** Properties of the carbon fibers (given by the manufacturer).

Carbon fibers content	100%, (100%)
Carbon content	94% (>92%)
Remaining sizing level	<0.1%
Density (Continuous fiber)	1.7 < d < 2.0 g/cm^3^
Mono filament diameter Median length	7 μm +/−2 100 μm +/−20
Tensile strength	3500 MPa
Elongation at break	1.5%
Young’s modulus (Tensile)	230 GPa

**Table 3:** Mix proportions for reference and GNP-composites.

Mixture	GNPs Type	w/c Ratio	Cement g	GNPs wt.%
Ref	-	0.5	470	-
N_0.05	N008-100-N	0.5	470	0.05
P10_0.05	N008-100-P10	0.5	470	0.05
P40_0.05	N008-100-P40	0.5	470	0.05
N_0.2	N008-100-N	0.5	470	0.2
P10_0.2	N008-100-P10	0.5	470	0.2
P40_0.2	N008-100-P40	0.5	470	0.2

**Table 4:** Mix proportions for reference and rCFs-composites.

Mixture	w/c Ratio	Cement g	rCFs wt.%
Ref	0.5	470	-
rCFs_0.1	0.5	470	0.1
rCFs_0.5	0.5	470	0.5
rCFs_1.0	0.5	470	1
rCFs_1.5	0.5	470	1.5
rCFs_2.0	0.5	470	2.0
rCFs_2.5	0.5	470	2.5
